# Ionizing Radiation and Translation Control: A Link to Radiation Hormesis?

**DOI:** 10.3390/ijms21186650

**Published:** 2020-09-11

**Authors:** Usha Kabilan, Tyson E. Graber, Tommy Alain, Dmitry Klokov

**Affiliations:** 1 Department of Biochemistry, Microbiology and Immunology, Faculty of Medicine, University of Ottawa, Ottawa, ON K1H 8M5, Canada; ushakabilan54@gmail.com; 2 Children’s Hospital of Eastern Ontario Research Institute, Ottawa, ON K1H 8L1, Canada; tyson@arc.cheo.ca; 3 Institut de Radioprotection et de Sureté Nucléaire (IRSN), PSE-SANTE, SESANE, LRTOX, 92262 Fontenay-aux-Roses Cedex, France

**Keywords:** ionizing radiation, protein synthesis, mRNA translation, low doses, radiation hormesis

## Abstract

Protein synthesis, or mRNA translation, is one of the most energy-consuming functions in cells. Translation of mRNA into proteins is thus highly regulated by and integrated with upstream and downstream signaling pathways, dependent on various transacting proteins and cis-acting elements within the substrate mRNAs. Under conditions of stress, such as exposure to ionizing radiation, regulatory mechanisms reprogram protein synthesis to translate mRNAs encoding proteins that ensure proper cellular responses. Interestingly, beneficial responses to low-dose radiation exposure, known as radiation hormesis, have been described in several models, but the molecular mechanisms behind this phenomenon are largely unknown. In this review, we explore how differences in cellular responses to high- vs. low-dose ionizing radiation are realized through the modulation of molecular pathways with a particular emphasis on the regulation of mRNA translation control.

## 1. Introduction

**Figure 1:**
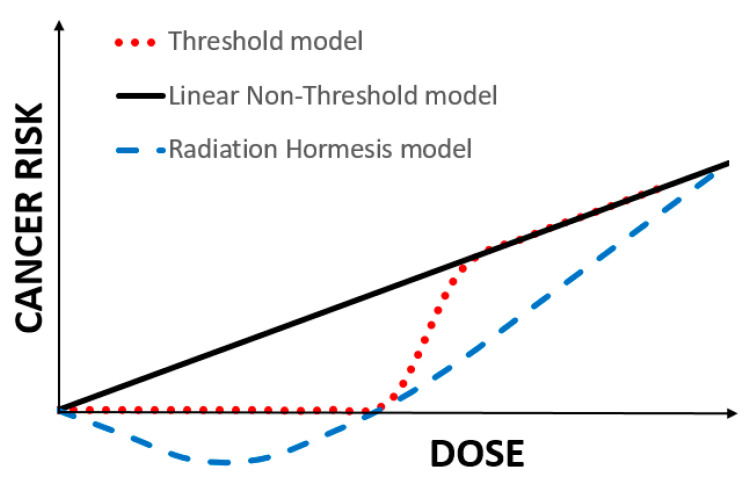
Schematic representation of the three main dose–response models linking cancer risk to dose of IR exposure. The Linear Non-Threshold (LNT) model states that cancer risk increases with dose in a linear fashion from a dose of zero units onwards. It is supported by human epidemiological data at intermediate to high doses, whereas supporting evidence in the low dose region (<100 mGy) is weak. The LNT is a foundation of the regulatory policies put forth by the International Commission on Radiological Protection. The Threshold model implies the existence of a threshold dose, below which no measurable increase in cancer risk is observed. The radiation hormesis model assumes a negative cancer risk at low-dose exposures, resulting in protection from cancer. The radiation hormesis model is supported by a large body of radiobiological literature showing beneficial responses to LDR, including *in vivo* animal cancer and mortality studies.

Ionizing radiation (IR) is ubiquitous in the Universe and on Earth and is able to elicit biological response due to the ionization of molecules in the cell. Humans are exposed to IR from both natural and anthropogenic sources, such as medical diagnostic or therapeutic procedures, nuclear power generation, or military and industrial nuclear applications. The amount or dose of IR, measured in Gy or Sv (1 Gy = 1 Sv for most common radiation types, such as γ-radiation, X-rays and β-particles), received by a person determines the biological outcome of such exposure. Due to its ability to damage DNA at high doses, IR is considered a carcinogen [1] and epidemiological studies support dose dependent increases in cancer risk above 100 mGy [2,3] (Figure 1). However, a person receives an annualized average of 2.4 mSv from various sources, including background radiation [4]. This easily falls into the range of low-dose IR (LDR) defined as <100 mGy [5]. Remarkably, although there are habitable areas on Earth where natural background radiation is orders of magnitude higher, no increased health detriment has been documented for those human populations [6,7]. Medical diagnostic procedures incorporating computed tomography (CT) scans have been a growing contributor to the annual dose over the last three decades in developed countries, causing concerns over potentially increased risks of cancer [8]. Yet, while CT scans deliver doses 2–3 orders of magnitude higher than a standard chest X-ray, an individual would require several per year to reach the 100 mGy LDR range limit.

In contrast, radiotherapy procedures deliver orders of magnitude higher doses compared to diagnostic imaging [9]. Those mostly include radiotherapy of cancer and account for 40–50% of all cancer patients in the world [10]. Whereas the capacity of IR to damage and kill cells is the key feature of cancer radiotherapy regimens, it is believed they also increase chances of neoplastic transformation of normal cells peripheral to the tumor upon exposure to gradients of radiation dose. However, association between LDR exposure and cancer incidence is controversial and lacks experimental support [11,12,13,14,15]. Moreover, numerous studies have documented stimulatory and beneficial effects of exposure to LDR, collectively called radiation hormesis, in a variety of experimental models [16,17]. The debate surrounding the effects of LDR highlights the fact that substantial knowledge gaps exist in our understanding of the molecular mechanisms that govern biological responses and health outcomes upon exposure to LDR.

Cellular responses to external stimuli, including stressors such as IR, are realized via changes in post-translational modifications, transcription and translation to ensure repair, survival and homeostasis by engaging various evolutionary conserved molecular signaling and defense circuits [18,19,20]. Depending on the amount of damage sustained by a cell after exposure to IR and its ability to cope with this damage, various choices are made to control the propagation of DNA mutations into future cell populations and hence, in the long run, the disease risk [21]. Master switch regulators, such as the tumor suppressor p53 protein, facilitate these transitions between cell cycle arrest, DNA repair, apoptosis and survival [22]. Various mechanisms of translation control, including by p53, play important roles in the execution of these complex programs [23,24]. Interestingly, as seen in other “stressful” cellular contexts, transcriptional IR-dependent stress response profiles correlate relatively poorly with translational or proteomic profiles, highlighting an important role of the control of protein synthesis in defining the ultimate repertoire of proteins that execute stress response programs [25]

Upon exposure to LDR, which typically produces low levels of DNA damage representing no threat to the survival of cells, an enhanced functioning of various defense mechanisms has been demonstrated. This includes the activation of DNA repair [26,27,28] and antioxidant [29,30] pathways, cell cycle regulation [26,31] metabolism [32], as well as crosstalk between these various mechanisms [33,34]. Although early work has shown that LDR-dependent resistance to stress requires *de novo* protein synthesis [35,36], the involvement of translation control in the variety of specific molecular defense mechanisms triggered by LDR has not been tested experimentally. In contrast, some empirical data exist on the effects of high-dose IR (HDR) exposure on mRNA translation. Since molecular and cellular responses to LDR vs. HDR are different not only in magnitude but also in specificity [37,38,39,40], we sought to review the current knowledge on the role of translation control in these responses to IR with a particular focus on a potential link to radiation hormesis.

## 2. Radiation Hormesis

### 2.1. Radiation Hormesis and Dose-Response Considerations

The term radiation hormesis introduced in the mid-20th century refers to a variety of biological responses to low (and sometimes intermediate; 100–500 mGy) doses of IR that can be characterized as stimulatory and protective (at either the cellular or organismal levels) [41,42]. These effects, however, have been well documented much earlier. For example, in 1919, an increase in the lifespan of the flour beetle *Tribolium confusum* following exposure to low doses of X-rays was reported [43]. Scientific literature on radiation hormesis is extensive and suggests it is a universal biological phenomenon found across the phyla [44] (see Supplementary Materials Table listing species/models where radiation hormesis has been observed). Hormesis is not limited to IR-dependent stress as it has been extensively observed upon exposure to low doses of a variety of genotoxic stressors, including chemical agents [45,46,47,48] and heat shock [49,50,51]. Certainly, hormesis can even form an integral part of homeostatic systems; in the brain the excitatory neurotransmitter glutamate facilitates synaptic transmission, yet a dose that is too high can lead to apoptosis (a phenomenon called excitotoxicity). Transgenic mice that overexpress *Glud1* have small but significant increases (10–15% over wildtype) in brain glutamate concentrations throughout life and are more likely to release glutamate at depolarized synapses. These mice exhibit smaller infarcts and less edema compared to their wildtype counterparts following ischemia-reperfusion injury, suggesting that the normally toxic glutamate has a protective effect for the brain at this superphysiological, yet subtoxic, dose [52]. This conspicuous body of evidence suggests hormetic stressors target ancient and highly conserved cellular pathways. Furthermore, hormesis, including radiation hormesis, has been acknowledged as an important factor in the evolution of life [53,54]. Indeed, background radiation has been much higher than the present day in the various periods of life history [55] and may have played a significant role in generating cellular adaptations that overcome the continuous pressure of low and mild genotoxicity. As a result of such selective pressure, cells would have developed diverse and complex defense mechanisms consisting of interacting networks of signaling and metabolic pathways making them fit to effectively cope with LDR exposures [55,56].

In striking contrast to the clear genotoxic and carcinogenic effects observed at high doses, and in spite of a large number of studies demonstrating radiation hormesis in a variety of contexts, this phenomenon has not found widespread acceptance in the scientific community and has not been accounted for in the evaluation of human health risks upon exposure to LDR [57]. Instead, the international system of radiological protection that regulates human exposure to IR [58] is based on an overarching assumption that has become known as the Linear-Non-Threshold (LNT) model. The LNT model postulates that the risk of cancer incidence increases linearly with dose irrespective of how low the dose may be (Figure 1) [58]. The linearity of such a dose–response curve observed almost a century ago for mutations in *Drosophila melanogaster* germ cells upon high-dose X-ray exposure became the primary justification for the adoption of the LNT model for human cancer risk estimates [59]. Further evidence supporting the LNT model comes predominantly from epidemiological studies of human cohorts exposed to IR, such as the Japanese atomic bomb survivors, nuclear workers or residents of the Techa river area [2,60,61]. However, different statistical analyses of the same cohorts [62,63] or military personnel involved in US nuclear bomb tests [64] produce antipodal conclusions with some showing no significant cancer risk at low doses of IR. Cancer, however, is a complex disease that initiates and progresses in a multistep manner and requires bypassing multiple defense mechanisms present in cells and organisms [65]. Thus, applying a universal, linear correlation of the number of radiation-induced DNA lesions to cancer incidence in a given population is problematic. In addition, several *in vivo* and *in vitro* radiobiological studies support conclusions that are inconsistent with the LNT model [66,67,68,69,70,71,72,73]. Nonetheless, the implementation of the LNT model (the most conservative compared to the threshold or radiation hormesis models—Figure 1) in radiation risk management has resulted in strict radiation-related policies, including in medical imaging and diagnostics [74] and has been criticized as causing a greater detriment to public health than radiation exposure itself [75]. This continuous debate exemplifies the need for more studies on the biological effects of LDR.

### 2.2. Radiation Adaptive Responses

Radiation adaptive (or radioadaptive) response refers to an acquired resistance to highly damaging HDR as a result of pre-exposure to LDR and represents a particular case of radiation hormesis [71,76]. Since studies of radiation hormesis are typically descriptive in nature and do not offer an insight into underlying cellular and molecular mechanisms—justifying critique and dismissal from regulatory policies—radioadaptive response studies provide an opportunity for better mechanistic understanding. HDR exposure is known to produce readily detectable biological outcomes, including DNA strand breaks, chromosomal aberrations, cell or organism killing and tumorigenesis. Thus, any protective response from HDR insults conferred by LDR pre-exposure could report hormetic effects that may otherwise be undetectable after LDR exposure alone. Although numerous studies have demonstrated radioadaptive responses of organisms *in vivo* [77], we will focus predominantly on the more mechanistic *in vitro* evidence. This will draw a clearer link between LDR-triggered molecular responses to mRNA translation control.

Cellular toxicity and genotoxicity are typical endpoints in the studies of the radioadaptive response. Olivieri et al [71] pioneered radioadaptive response studies by showing in 1980 that human peripheral blood lymphocytes cultured with low concentrations of tritiated thymidine (a source of β-radiation) developed fewer chromosomal aberrations when subsequently exposed to 1.5 Gy of X-rays compared to cells that were not pre-exposed to low doses. These findings of LDR-conferred resistance to cytogenetic damage, which can be a result of the enhanced repair of DNA breaks or other types of genomic instability, were later also reported in a variety of cell types and models [73,78,79,80,81]. The involvement of DNA repair mechanisms in the radioadaptive response has been examined and produced mixed results. Some studies confirmed the role of various DNA repair pathways in LDR-induced resistance to high-dose insults [82,83,84,85,86], whereas other studies found no effect of LDR on DNA repair [87,88]. It appears that radioadaptive responses may be influenced by a number of factors ranging from radiation characteristics to cell type and/or genetic makeup [89,90]. However, the idea that early response mechanisms triggered by LDR exposure and mediating radioadaptation, are related to the DNA damage response (DDR) has found broad acceptance [91,92]. DDR refers to a series of complex signaling networks that first sense DNA lesions and then initiate and coordinate adequate responses that can result in damage repair and survival, or conversely, apoptotic elimination of a severely damaged cell [21].

The first line of defense from acute high-dose IR stress that is regulated by DDR includes activation of not only DNA repair mechanisms, but also detoxification of reactive oxygen species [93,94,95]. In fact, this enhanced antioxidant function has often been associated with the beneficial effects of LDR exposure. In quiescent normal human fibroblasts, exposure to 10–100 mGy of γ-radiation was shown to upregulate cellular antioxidant capacity by ectopic overexpression of MnSOD, catalase and glutathione peroxidase, and by the suppression of superoxide anion generation through flavin-containing oxidases [96]. Activation of the same enzymes was observed in human peripheral blood mononuclear cells primed with 100 mGy and challenged with a high dose of 2 Gy [97], and a greater number of antioxidant enzymes were reported in human lymphoblastoid cells primed with 20 mGy and challenged with 3 Gy [98]. In the former study, the enhanced activity of the antioxidant enzyme was mediated by increased nuclear translocation of the transcription factors Nuclear factor E2-related factor 2 (NRF2) and Nuclear factor kappa B (NF-κB)—known regulators of antioxidant circuits—providing mechanistic insight into the radioadaptive antioxidative response. NRF2 was also found to be implicated in the response to LDR in human lung cancer cells [99]. The involvement of antioxidant defense mechanisms in the radioadaptive response was further supported by several in vivo mouse studies [100], including one carried out in wild rodent species inhabiting the radio-contaminated areas in the Chernobyl accident exclusion zone [101]. Tumor necrosis factor alpha (TNFα) signaling via TNFR1 and TNFR2 receptors was shown to be implicated in the regulation of SOD2 upregulation and resulting protection from chromosomal damage, constituting a radioadaptive response in mouse fibrosarcoma cells [30]. Interestingly, the MnSOD protein produced in LDR-exposed normal human skin keratinocytes interacted preferentially with a set of proteins involved in the regulation of mitochondrial metabolism, DNA repair and apoptosis, suggesting that a crosstalk between various cell defense mechanisms plays a crucial role in response to LDR [34].

**Figure 2:**
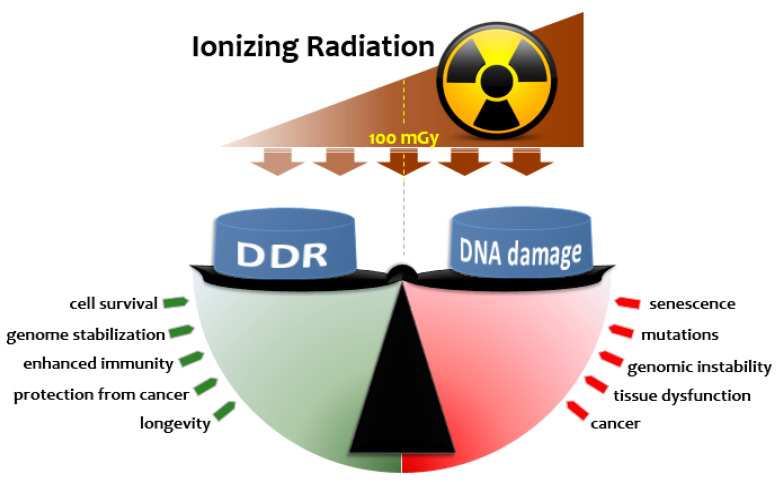
A dynamic interplay between the amount of DNA damage and DNA damage response (DDR) upon exposure to IR determines the biological outcome in cellular and organismal contexts. Initial DNA lesions caused by exposure to IR are proportional to dose and trigger the DDR; a signaling cascade that senses damage and activates various DNA repair mechanisms, cell cycle arrest, if required, antioxidant defense and other relevant pathways. The magnitude of DDR and downstream branching to more specialized pathways (e.g., survival vs. apoptosis or homologous recombination [HR] vs. non-homologous end joining [NHEJ] DNA repair) may depend on various factors, such as dose, dose rate, radiation type and linear energy transfer, cell type and, microenvironment. Upon exposure to LDR, the DDR triggered is thought to not only repair the low amount of DNA damage caused, but also to render cells resistant to subsequent genotoxic stresses (a radioadaptive response). Such LDR-induced adaptation may last long enough to suppress the rates of mutation, genomic instability, senescence/aging and tumorigenesis caused by either HDR or endogenously generated reactive oxygen species, resulting in radiation hormesis. If, however, the degree of DNA damage produced by IR is high enough—typically above a certain threshold dose that may vary depending on cell type/organism—the capacity of the triggered DDR is insufficient to complete repair. This causes detrimental consequences, such as mutations, genomic instability, neoplastic transformation or tissue dysfunction. The interplay between the DDR and DNA damage is, therefore, dynamic and depends on a multitude of contextually determined factors.

Coordination of the various signaling pathways during DDR triggered by radiation is at the core of defining the net result relevant to long-term outcome. Various candidates have been suggested to orchestrate this coordinated response to LDR, including p53 [102] and NRF2 [103], and involving major signaling pathways such as ATM/ERK/NF-κB [26], PKC-p38MAPK-PLC [86], AKT/ERK [79], TNFα [104], FOXO3A [105] and TGF-β [106]. This coordination and execution of the programs can be realized at transcriptional [107], translational [23] and post-translational levels [108]. It appears that the balance between sustained damage and the elicited DDR signals plays a key role in shaping the physiological outcome (Figure 2). Upon LDR exposure, DNA damage would not exceed the capacity of the baseline or LDR-induced repair mechanisms, resulting in not only survival, but also adaptation that can be characterized by enhanced protective mechanisms. This adapted state has been shown to last long periods of time ranging from weeks to months after the triggering stimuli in the form of genome stability (11 months in mice [109,110]) and enhanced immune function [107,111] that may contribute to better protection from cancer [112,113,114] and also longevity [115,116,117]. If, however, the amount of DNA damage exceeds a certain threshold level, the cell may not be able to repair all damage or repair it properly, leading to genomic instability, senescence, altered function and disease. While this threshold in repair capacity appears to vary depending on the cell type and age (i.e., it is genomically- or epigenomically-encoded), radioadaptation provides an elegant mechanism for DDR plasticity—allowing its setpoint threshold to change over the lifetime of a cell depending on its environmental context.

## 3. Translation and IR Stress Responses

**Figure 3:**
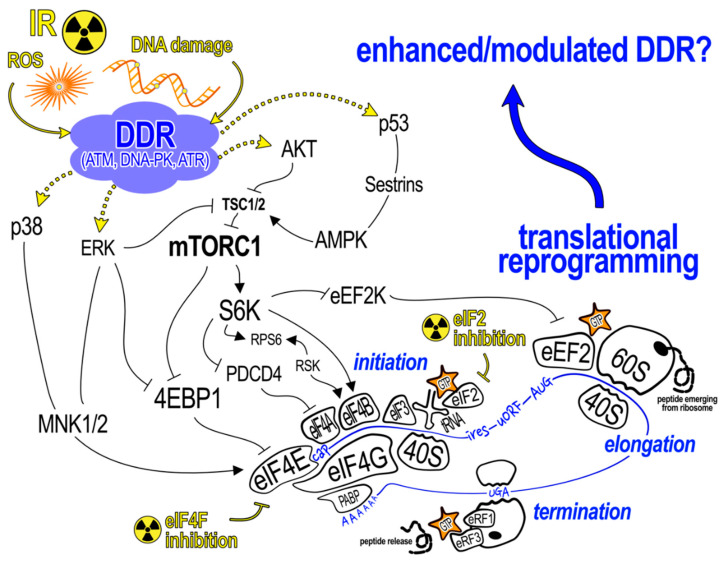
Cellular signaling pathways and molecules that might be involved in reprogramming mRNA translation following exposure to IR. Reactive oxygen species (ROS) and/or DNA damage produced as a result of high doses of IR are known to activate the DDR consisting of highly interconnected kinase cascades (ATM, DNA-PK, ATR). Stimulation of these pathways converges on known signaling cascades that regulate mRNA translation. The major steps of translation shown towards the bottom of the diagram in blue. Note that all 3 steps require energy in the form of GTP to nucleate the ribosome (made up of 40S and 60S ribosomal subunits) on the mRNA (initiation), to elongate the peptide chain (elongation) and to release the completed peptide (termination). Translation control is exerted largely at the initiation step and specifically by modulating the formation of the eIF4F cap-binding complex and/or activity of eIF2 through phosphorylation of its alpha subunit (eIF2α). Sequence motifs within mRNAs (shown in blue font along the mRNA and defined in the text) have different sensitivities to these control points, allowing the finely-tuned regulation of single species and/or groups of mRNAs. Radioresistant phenotypes (e.g., an enhanced DDR) could be mediated by translational reprogramming resulting from stimulation of these upstream pathways following LDR exposure.

mRNA translation, along with replication and transcription, is a fundamental and evolutionary conserved cellular process that is absolutely required for life. Protein synthesis is the most energy-expensive of all cellular processes. In the bacterial cell, ~50% of the energy is consumed by translation, while in mammalian cells, it is 30% [118]. In eukaryotes, translation of an mRNA into protein is accomplished in 3 major steps: initiation, elongation and termination. The limiting step is initiation which, together with its complexity, offers a variety of ways for controlling translation to rapidly save energy if needed [119]. In classical cap-dependent translation initiation, recruitment of the 40S small ribosomal subunit to the mRNA to be translated is facilitated by the heterotrimeric eukaryotic initiation factor (eIF) 4F complex, comprising eIF4E, eIF4G, and eIF4A, which is anchored through eIF4E at the methylguanosine cap structure at the 5′ end of the mRNA (Figure 3). Poly (A) binding protein (PABP), which interacts with the 3′ end of a poly(A)+ mRNA can also interact with eIF4F, thus forming a mRNA loop which is thought to enhance translation. The 40S subunit bound to eIF3—which prevents subunit joining—is also bound to a ternary complex consisting of eIF2 bound to GTP- and initiator methionyl-charged tRNA, forming the 43S pre-initiation complex (PIC). This triggers the 5′—3′ scanning of the 43S PIC along the 5′ untranslated region (UTR) of the mRNA in search of an AUG start codon in the correct context. Recognition of a start codon then prompts the release of initiation factors and binding of the 60S ribosome subunit followed by translation elongation and termination which produces a completed peptide [119].

Under stress conditions, such as IR exposure, different branches of DDR signaling can alter translation initiation at several key steps. When cells experience severe DNA damage (e.g., following exposure to 1 Gy and higher IR doses) the immediate cellular response is to redirect all resources to alleviate the stress and repair damage. This requires arresting the cell cycle and shutting down all functions that may increase cellular stress. Given the high energy cost of mRNA translation, the transient inhibition of global protein synthesis is a highly conserved cellular response to stress [120]. A major effector of this response is phosphorylated eIF2 which is a strong competitive inhibitor of the eIF2-GTP-tRNA ternary complex, required for translation initiation. Several mammalian protein kinases such as GCN2, PERK, HRI and PKR, which are activated in response to both extrinsic and intrinsic stressors, can phosphorylate eIF2 [121].

However, it is another kinase that has arguably a greater role to play in regulating translation initiation in the context of IR. The mechanistic target of Rapamycin (mTOR) coordinates cellular growth and DNA repair in response to environmental stresses, is a serine/threonine protein kinase belonging to the PI3K kinase family and exerts its functions as a central controller of major cellular processes by forming two unique complexes: mTORC1 and mTORC2 [122]. mTORC1 integrates upstream signaling from the DDR and acts as a gatekeeper to mRNA translation, while the exact role of mTORC2 in these processes remains nebulous (Figure 3). The mechanism of global suppression of translation upon genotoxic stress seems to be mediated by a p53-dependent induction of Sestrin1 and Sestrin2 followed by an activation of AMPK and its downstream target TSC2, resulting in an inhibition of mTORC1 [123]. Dependence of mTORC1-mediated translation control upon a p53-mediated genotoxic stress response is important since cancer cells are notorious for p53 loss-of-function mutations. Furthermore, Braunstein et al. demonstrated that p53-dependent global suppression of translation is mediated by a competitive interaction of hypophosphorylated 4E-BP1 with eIF4E, reducing its availability for cap-dependent translation initiation, and is preceded by an early and transient stimulation of protein synthesis to enhance DDR, also in an mTOR-dependent manner [124]. This regulatory circuit can, therefore, be regarded as an IR-induced homeostatic pathway present in nontransformed breast epithelial cells that facilitates proper DDR. Critically, this innate response to radiation was lost in transformed breast cancer cells. The same group later identified the mRNA substrates in this translational response to IR which we will discuss further on in the text [125]. A more recent study using a dual mTORC1/2 small molecule inhibitor, INK128, has strengthened the connection between IR, DNA double-strand break (DSB) repair, and mRNA translation. Interestingly, normal human fibroblasts, which, as expected, are more sensitive to cell death at lower doses of IR than transformed cells, showed no increased radiosensitivity at these lower doses following treatment with INK128 [126]. Moreover, an INK128-dependent increase in γH2AX foci seen in the transformed cell line was absent in the nontransformed counterpart [126], likely due to the decreased requirement for eIF4E (and thus mTOR) in normal cells [127]. Taken together, data from these studies suggest that mTOR in nontransformed cells acts as a permissive gate; its activation is necessary, but not sufficient for the translation of specific sets of mRNAs following IR, whose encoded proteins function in DNA repair, and which may mediate a radioprotective phenotype (these studies did not formally test this using a radioprotective experimental model). Additional IR-dependent signals that specify which subsets of radioprotective mRNAs are ultimately translated remain to be elucidated.

Translation control provides a means to very quickly, within minutes, increase amounts of proteins that are most important for a proper response to genotoxic stress. This is implemented by redeployment of the translation machinery to functional groups of existing mRNAs, instead of activating their transcription [119]. Specific regulation of mRNA subsets in response to IR, when global, cap-dependent protein synthesis might be transiently suppressed, could be achieved by cap-independent mechanisms of translation initiation. One such mechanistic model invokes cis-sequence elements within subsets of mRNAs called internal ribosome entry sites (IRES) which act to recruit ribosomes to an mRNA downstream of the 5′ cap. Earlier studies using cDNA microarrays that quantified transcripts present in polysomes in conditions of limiting eIF4F complex estimated internal ribosome entry to be used by ~10% of cellular mRNAs [128]. A similar percentage was observed in a more recent unbiased and elegant approach by Weingarten-Gabby et al [129]. Importantly, many of the genes involved in stress responses have been found to contain IRES in their mRNAs, including the master switch regulator p53, anti-apoptotic Bcl-2, BAG-1 and XIAP [130,131,132], and many DNA repair factors and cell cycle regulators [125,133]. These mRNAs can, therefore, be preferentially translated upon genotoxic stress which, on a background of globally suppressed cap-dependent translation, would result in the synthesis of proteins required for DNA repair, cell cycle arrest and survival [23]. The molecular mechanisms by which IRES confer recruitment of ribosomes are quite heterogeneous, with protein transacting factors (ITAFs) thought to act as both adaptor proteins and RNA chaperones in many different combinations [128]. Furthermore, with the discovery of other cap-independent initiation mechanisms, it is becoming clear that “cap-independent” is not synonymous with IRES-mediated translation [134]. Complicating matters considerably is a differential requirement of mRNA species for eIF4E and other factors in cap-dependent initiation. For instance, highly invasive breast cancer cells overexpress eIF4G1, a key factor in the eIF4F complex, and this overexpression was shown to be required for radioresistance to selectively promote translation of mRNAs involved in DNA repair and survival, such as ATM, ATR, p53, 53BP1, MRE11, GADD45α, XIAP, BRCA1/2, Survivin, ATRIP, CHK1, PARP, RFC2-5 and HIF-1α [125]. Signaling and repair of DNA DSB was suppressed in these cells after the knock-down of eIF4G1. Yet, the reduction in eIF4E levels had only minimal effects on radiosensitivity, suggesting a lenient eIF4E requirement for these mRNAs; the precise mechanism of such regulation remains unclear [125].

Another key DNA damage signaling pathway is triggered by DNA-PK, and this seems to use yet another translation initiation mechanism that is activated during stress. Upon UVB exposure that induces DNA damage, DNA-PK-dependent repression of global translation, along with the specific upregulation of a subset of DNA repair mRNAs, was reported for human HeLa cells [135]. It is worth noting that these mRNAs were not overlapping with those translationally upregulated shortly after γ-radiation exposure [125]. Notably, the subset of mRNAs recruited to polysomes during UVB irradiation were enriched in upstream open reading frames (uORFs) present in their 5′ UTRs [135]. About half of mammalian mRNAs have at least one uORF in their 5′ UTR [136] and these elements have an important role in translation control under global repression conditions, such as DNA damage stress, exemplified by the control of the stress response transcription factor ATF4 [137]. Translation of uORFs is favored over that of the main, downstream ORF under normal conditions when eIF2α is active, while translation initiation shifts to the main ORF during stress when eIF2α is transiently phosphorylated [138]. Thus, it appears that the different spectrum of DNA lesions induced by UVB vs. γ-radiation—the former producing predominantly pyrimidine dimers and the latter DSB and single-strand breaks—can trigger specific DDR signaling pathways leading to measurably different mechanisms of translation control. Yet, DNA-PK does play an essential role in IR-induced DDR signaling to activate cell cycle checkpoint and facilitate the DSB repair pathway by non-homologous end joining (NHEJ), and so it is important to understand that many of these initiation mechanisms are not mutually exclusive and can operate in a concerted fashion both between and within transcripts [139].

In an attempt to unify a variety of mRNA translation control mechanisms activated in response to changing environmental conditions, Keene and Tenenbaum proposed a post-transcriptional operon model that places RNA-binding proteins (RBPs) at its core [140]. These proteins can operate in a combinatorial manner binding to specific sequences in mRNAs that belong to a common functionally related group, such as DNA repair, cell cycle arrest, apoptosis. These interactions can then affect various steps of post-transcriptional gene expression, including mRNA splicing, transport, stability and translation. Consistent with this concept, IR-induced activation of the ATM-CHK1/2 DDR signaling axis can lead to phosphorylation of Human antigen R (HuR), a prominent regulator of mRNA stability, splicing and translation via interaction with specific sequences found in the 3′ UTR of many transcripts [141,142]. In human lymphocytes exposed to 1 Gy of IR, ATM-dependent phosphorylation of HuR was reported to facilitate its binding to a subset of mRNAs [143]. The disruption of IR-induced binding of HuR to mRNA enhanced cell killing, suggesting an important physiological role of the ATM-CHK1/2-HuR signaling in response to IR stress [144]. Other RBPs have also been identified that are regulated not only by ATM, but also by DNA-PK [145,146,147,148]. Furthermore, larger networks of proteins involved in post-transcriptional gene expression control were identified as effectors of both ATM [149] and DNA-PK [150]. Systems biology approaches will be required to identify these complex translation regulation patterns elicited in response to IR exposure and their driving signaling cascades. Such research is necessary to further develop post-transcriptional operon models and to understand the complexity of multiple layers of translation control and their role in IR exposure outcomes, such as cancer or hormesis.

## 4. Translation Control in Response to LDR 

The requirement for *de novo* protein synthesis for LDR-induced protective responses has been demonstrated in early studies investigating radioadaptive responses [35,36]. These studies showed that human lymphocytes exposed to 10 or 50 mGy can become resistant to a subsequent high challenging dose of IR, but importantly, this effect is negated by cycloheximide treatment in between the two irradiations [35,36]. The authors found that LDR pre-exposure prevented chromosomal aberrations that occurred as a consequence of unrepaired or misrepaired DNA strand breaks from the high IR dose. Since the time window for this protection and its abrogation by cycloheximide was 0-6 h after the LDR priming, these findings propose direct evidence that selective translation control of mRNAs involved in DDR is required to elicit such hormetic effects. Nonetheless, detailed dissections of translation control during LDR and/or in an experimental model of radioprotection have not yet been performed. Instead, we present additional studies that have linked cellular responses to radiation and control of protein synthesis.

As reviewed in the previous section, ATM- and DNA-PK-dependent signaling, that take place on challenge with high-dose IR can lead to reprograming of translation, such that a subset of functionally relevant (e.g., those involved in DDR) transcripts are translated more efficiently [25] while global translation is inhibited by restricting availability of the components of the classical cap-dependent translation initiation complex [20]. Sensing and repair of IR-induced DSB was altered by lowering levels of the eIF4G1 factor, therefore making it a possible candidate for mediating LDR-induced adaptation [125]. Among the targets of eIF4G1-mediated translation activation post-IR is BRCA1, a tumor suppressor that facilitates error-free homologous recombination (HR) DSB repair and that causes breast cancer in humans when mutated [151]. Remarkably, BRCA1 was shown to have a noncanonical role in regulating translation of a large number of transcripts enriched for cellular functions related to DNA repair, cell cycle arrest, apoptosis and other DDR-relevant signaling [125]. Furthermore, BRCA1 was shown to effect its regulatory function by binding to mRNA in a PABP1-dependent manner [151,152]. Shifting a balance from the error-prone NHEJ towards the error-free HR DSB repair pathways that can be regulated by 53BP1, including via eIF4G1-mediated translation control [125], has been proposed as one of the potential responses to chronic LDR exposure in primary human mesenchymal stem cells [153], human fibroblasts [75] and myoblasts [154]. Therefore, it is feasible to suggest that a translational reprogramming of DSB signaling and repair is involved in LDR responses that lead to enhanced ability to cope with either exogenous (e.g., genotoxic environmental stresses) or endogenous (e.g., oxidative metabolism and replication errors) DNA damage. This state of enhanced genome stability, if maintained for long periods of time, may lead to radiation hormesis at the cellular and organismal levels and be expressed as lower incidence of neoplastic transformation [81], suppressed tumorigenesis [112] or extended lifespan [116].

Translational reprograming observed in cells upon heat or cold stress may provide additional mechanistic insight into putative translation control from exposure to LDR. Mild heat stress has been shown to trigger an adaptive response that protects cells and organisms against HDR in much the same way as an LDR pre-exposure [69,155]. Heat shock proteins induced by LDR exposure—in particular, Hsp70—have been implicated in radioadaptive responses in human cells *in vitro*, as well as in mice and fruit flies *in vivo* [156,157,158]. It appears that heat shock proteins can be induced in a variety of stress conditions and have a prominent role in adaptation to such influences [159]. At the same time, production of heat shock proteins can be regulated at the translation level [23]. As mentioned earlier, global repression of translation observed with many different cellular stressors is accompanied by the selective translation of a subset of mRNAs including heat shock proteins Hsp70, Hsp40, Hsp60, Hsp90, Hsp100 and Hsp27. This translation control is thought to be realized predominantly via specific RBPs and/or ITAFs, and a limited requirement for eIF4E in translation initiation has also been documented [23]. Therefore, the translational control of heat shock proteins that are potent effectors of LDR-induced responses represents a viable mechanism of linking radiation hormesis and translation control.

Lastly, beneficial effects of LDR on cellular [63] and organismal aging [100,160] offer an exciting opportunity for linking these effects to mTOR-mediated translation regulation. mTOR has a prominent role in modulating aging, longevity and aging-related health disorders [161]. In particular, attenuated activity of mTOR and related protein synthesis is a key factor in lifespan and/or health span extension by rapamycin or caloric restriction [161,162]. At the cellular level, this can be at least partially explained by the diminished accumulation of proteotoxic and oxidative stress products [122,163]. As we have seen in this review, a major effector of mTOR regulation is translation, and although direct experimental evidence linking LDR-induced suppression of aging with mTOR-mediated translational reprograming has not yet been reported, such a link appears logical. In fact, research in this area is one of the priorities of our group using normal human lung fibroblasts as a cellular model for LDR-induced suppression of senescence. It will be intriguing to test whether a delay of cellular senescence commonly seen in this cellular model upon exposure to LDR may be associated with modulated translation, and it will be important to identify mechanistic drivers of such reprograming.

## 5. Summary and Future Perspectives

Largely nonconcordant transcriptional and translational profiles of cells exposed to IR suggest that translation control acts as a gatekeeper, ensuring homeostatic plasticity in the synthesis of a protein repertoire that is essential for a proper cellular response. Such a response must include damage repair and genomic stabilization and represents the mechanism of adaptation to environmental stress, a fundamental property of life. Mild stress incurred by LDR, typically encountered environmentally, occupationally or during medical diagnostic procedures, is likely to trigger these protective mechanisms, leading to biological benefits, called radiation hormesis. This effect has been observed experimentally using a variety of models and endpoints, and using phenotypic metrics such as genomic stability, anticancer immunity and lifespan. Four lines of evidence support the hypothesis that translational control could mediate radiation hormesis: (1) direct experimental evidence showing that blocking *de novo* protein synthesis with small molecule inhibitors blocks the hormesis effect in irradiated cells; (2) detailed molecular dissections from HDR studies highlight the importance of translational control in these contexts; (3) substantial overlap and interplay of the translation machinery with molecular players in DNA damage signaling suggests intimate links with radiation hormesis; (4) the ancient translation machinery has been subject to constant evolutionary pressure caused by the presence of natural background radiation for hundreds of millions of years; thus, it is a likely player in the expression of the radiation hormesis phenotype.

Further research is warranted to directly demonstrate such involvement, such as in LDR-induced suppression of cellular senescence, which is characterized by substantial reprograming of protein synthesis under the control of mTOR signaling. Recent technological strides, availability and affordability of high-throughput genome-wide screening should aid in the identification of LDR-specific translatome profiles and their relevance to effecting radiation hormesis, especially in the context of long-term health related outcomes such as cancer and aging. Certainly, the multimodal integration of data using systems biology approaches will facilitate the understanding of the complex and multilayered network of factors that may link translation control to the potential hormetic response to LDR.

## Supplementary Materials

Supplementary materials can be found at https://www.mdpi.com/1422-0067/21/18/6650/s1.

## Abbreviations

53BP1	P53 binding protein
AKT	Protein kinase B
AMPK	Adenosine monophosphate-activated protein kinase
ATF4	Activating transcription factor 4
ATM	Ataxia telangiectasia mutated
ATR	Ataxia telangiectasia and Rad3-related
ATRIP	ATR interacting protein
Bcl2	B-cell lymphoma 2
BRCA	Breast cancer
CHK	Checkpoint kinase
Clud1	Glutamate receptor delta-1
CT	Computed tomography
DDR	DNA damage response
DNA-PK	DNA-dependent protein kinase
DSB	Double-strand break
eIF	Eukaryotic initiation factor
ERK	Extracellular signal-regulated kinase
FOXO3A	Forkhead box O-3 A
GADD45α	Growth arrest and DNA damage inducible alpha
GCN2	General control nonderepressible 2
GTP	Guanosine triphosphate
HDR	High-dose ionizing radiation
HIF-1α	Hypoxia inducible factor 1 alpha
HR	Homologous recombination
Hsp70	Heat shock protein 70
HuR	Human antigen R
IR	Ionizing radiation
IRES	Internal ribosome entry sites
ITAF	Initiation trans-acting factors
LDR	Low-dose ionizing radiation
LNT	Linear-non-threshold
MAPK	Mitogen-activated protein kinase
MRE11	Meiotic recombination 11
mTOR	Mechanistic target of Rapamycin
mTORC	Mechanistic target of Rapamycin complex
NF-κB	Nuclear factor kappa B
NHEJ	Non-homologous end joining
NRF2	Nuclear factor E2-related factor 2
ORF	Open reading frame
PABP	Poly (A) binding protein
PARP	Poly (ADP-ribose) polymerase
PI3K	Phosphatidylinositol 3-kinase
PIC	43S pre-initiation complex
PKC	Protein kinase C
RBP	RNA binding protein
RFC2-5	Replication factor C subunit 2-5
TGF-β	Transforming growth factor beta
TNFR1	Tumour necrosis factor alpha receptor 1
TNFα	Tumour necrosis factor alpha
TSC2	Tuberous sclerosis complex 2
UTR	Untranslated region
XIAP	X-linked inhibitor of apoptosis
