# Supplementary material for: Ionizing Radiation and Translation Control: A Link to Radiation Hormesis?
Source: Int J Mol Sci. 2020 Sep 11;21(18):6650. doi: 10.3390/ijms21186650 (PMC7555331; doi:10.3390/ijms21186650)
Supplement: Supplementary file 1 [file ijms-21-06650-s001.pdf]

Manuscript *Kabilan et al.*

Supplementary Table. A brief summary of radiation effects related to radiation hormesis observed in a variety of organisms/cell types.

Model organism	Range of dose/dose-rate	Effects	Reference
House Cricket (*Acheta domesticus*)	0.5-2 Gy	Survival fitness and reproductive maturity was observed at low (but not high) doses.	[1]
Rat (*Rattus norvegicus*)	0.75 Gy	γ- (but not X-ray) radiation promoted dermal wound healing; resistance to longitudinal stress.	[2]
Large Japanese Field Mouse (*Apodemus speciosus*)	0.05-21 mGy/day	Increased spermatogenesis in the mice treated with low doses of γ-radiation. The highest dose rate 21 mGy/day lead to an increase in neoplastic cell death which was not observed at lower dose rates.	[3]
Large Japanese Field Mouse (*Apodemus speciosus*)	4.1-13.9 µGy/hour	At low doses/dose rates of γ-radiation, there were no detrimental effects of spermatogenesis observed in the exposed mice population at Fukushima Daiichi Nuclear Power plant.	[4]
Fruit Fly (*Drosophila Melanogaster*)	400 mGy (irradiated at the rate of 0.014 mGy/hr for 12 days)	Chronic exposure to 400 mGy of γ-radiation upregulated DNA repair genes resulting in radioadaptive mechanisms.	[5]
Mouse (*Mus musculus*)	25-500 mGy	The frequency of aortic atherosclerotic lesion formation reduced in $APOE^{-/-}$ mice irradiated at 25-50 mGy while the control group (unexposed $APOE^{-/-}$ mice) showed a gradual increase in the lesion formation with time (8 months).	[6]
Fish (*Danio rerio*)	1.4-8.4 mGy	Low apoptotic signals observed at doses below 2.8 mGy resulting in radio adaptive responses. Bystander effect was also observed.	[7]
Fish (*Danio rerio*)	4.4 mGy	Active clearance of apoptotic cells was observed in response to low-dose radiation.	[8]
Worm *C. elegans*	0.5-1000 mGy	Increased activity of 20S proteasome in worms treated with highest dose (1 Gy) while this was not observed at lower doses.	[9]

Mouse<br>(*Mus musculus*)	650 and 2000 mGy	At 650 mGy, photoreceptor apoptosis was reduced through upregulation of Prdx2 gene resulting in decreased neuro degradation in rd10 mice and inducing radiation mediated neurohormesis.	[10]
Mouse<br>(MC3T3-E1 cells)	500 mGy	Stimulation of osteoblast proliferation, enhanced wound healing.	[11]
Human<br>(HELF-104 cells)	0.01-2 Gy	A delay in cellular senescence with acute 30-50 mGy doses of γ-radiation.	[12]
Human<br>(CCD-986 sk)	2-10 mGy	Decreased aneuploidy of chromosomes 1 and 2.	[13]
Fish – Medaka<br>(*Oryzias latipes*)	0 - 5.88 Gy	Radioadaptive responses seen in fish pre-exposed to low doses. Communication of protective signals between irradiated and non-irradiated individuals as an evidence of bystander effect.	[14]
Bacteria<br>*Photobacterium Phosphoreum*	100 mGy	Treatment with 100 mGy of ionization radiation using tritium for 15 days did not induce any mutation in the bacterial genome. Bacterial luminescence was higher without tritium penetration into the bacterial cells.	[15]
Protozoan<br>*Paramecium tetraurelia*	0-50 mGy/year	The cellular proliferation is stimulated upon irradiation and this stimulatory effect declines at doses below 50 mGy/year.	[16]
Plant<br>*Arabidopsis thaliana*	0.1-10 Gy	No increase in homologous recombination frequency upon acute irradiation at 0.1 – 0.5 Gy. 5-6-fold increase in the the homologous recombination frequency upon chronic irradiation at >200 mGy.	[17]
Plant<br>*Tradescantia BNL 4430*	50 mGy	50 mGy of acute γ-irradiation upregulated Calnexin-1 gene, membrane protein functions as a chaperone in endoplasmic reticulum. Overexpression studies using *E. Coli* strain BL21 (DE3) increased the cellular viability which suggest that IR upregulating Calnexin-1 in plants to withstand the radiation associated stress.	[18]
Plant<br>*Stipa capillata*	196 mGy	Induction of genes such as Superoxide Dismutase (SOD2), Glutathione Reductase (GR), Peroxidases (POD), Catalase (CAT), Glucose-6-phosphate Dehydrogenase (G6PDH) is observed upon irradiation.	[19]
Protozoa<br>*Tetrahymena pyriformis*	0.4 – 2.7 mGy	Stimulation of growth of the organism.	[20]
Bacteria<br>*Synechococus lividus*	31 mGy/year	Cells irradiated with low doses showed stimulated growth rates and the stimulatory effect diminished as radiation dose increased.	[21]

Yeast<br>*Wangiella dermatitidis*	7 mGy	Radiation induced higher survivability rate in melanin containing yeast compared to melanin lacking yeast. This effect was due to enhanced DNA repair mechanisms and genome stability.	[22]

SUPPLEMENTARY REFERENCES:

1. Shephard, A.M.; Aksenov, V.; Tran, J.; Nelson, C.J.; Boreham, D.R.; Rollo, C.D. Hormetic effects of early juvenile radiation exposure on adult reproduction and offspring performance in the cricket (Acheta domesticus). *Dose-Response* **2018**, *16*, doi:10.1177/1559325818797499.

2. Jabbari, N.; Farjah, G.H.; Ghadimi, B.; Zanjani, H.; Heshmatian, B. Acceleration of skin wound healing by low-dose indirect ionizing radiation in male rats. *Kaohsiung J. Med. Sci.* **2017**, *33*, 385–393, doi:10.1016/j.kjms.2017.05.013.

3. Takino, S.; Yamashiro, H.; Sugano, Y.; Fujishima, Y.; Nakata, A.; Kasai, K.; Hayashi, G.; Urushihara, Y.; Suzuki, M.; Shinoda, H.; et al. Analysis of the Effect of Chronic and Low-Dose Radiation Exposure on Spermatogenic Cells of Male Large Japanese Field Mice (Apodemus speciosus) after the Fukushima Daiichi Nuclear Power Plant Accident. *Radiat. Res.* **2017**, *187*, 161, doi:10.1667/rr14234.1.

4. Okano, T.; Ishiniwa, H.; Onuma, M.; Shindo, J.; Yokohata, Y.; Tamaoki, M. Effects of environmental radiation on testes and spermatogenesis in wild large Japanese field mice (Apodemus speciosus) from Fukushima. *Sci. Rep.* **2016**, *6*, 1–8, doi:10.1038/srep23601.

5. Koval, L.; Proshkina, E.; Shaposhnikov, M.; Moskalev, A. The role of DNA repair genes in radiation-induced adaptive response in Drosophila melanogaster is differential and conditional. *Biogerontology* **2020**, *21*, 45–56, doi:10.1007/s10522-019-09842-1.

6. Mitchel, R.E.J.; Hasu, M.; Bugden, M.; Wyatt, H.; Little, M.P.; Gola, A.; Hildebrandt, G.; Priest, N.D.; Whitman, S.C. Low-Dose Radiation Exposure and Atherosclerosis in ApoE −/− Mice . *Radiat. Res.* **2011**, *175*, 665–676, doi:10.1667/rr2176.1.

7. Choi, V.W.Y.; Cheung, A.L.Y.; Cheng, S.H.; Yu, K.N. Hormetic effect induced by alpha-particle-induced stress communicated in vivo between zebrafish embryos. *Environ. Sci. Technol.* **2012**, *46*, 11678–11683, doi:10.1021/es301838s.

8. Yuen Ping Ng, C.; Han Cheng, S.; Ngok Yu, K. Effect of Photon Hormesis on Dose Responses to Alpha Particles in Zebrafish Embryos. *Int. J. Mol. Sci. Artic.* **2017**, doi:10.3390/ijms18020385.

9. Dubois, C.; Lecomte, C.; Ruys, S.P. dit; Kuzmic, M.; Della-Vedova, C.; Dubourg, N.; Galas, S.; Frelon, S. Precoce and opposite response of proteasome activity after acute or chronic exposure of C. elegans to γ-radiation. *Sci. Rep.* **2018**, *8*, doi:10.1038/s41598-018-29033-1.

10. Otani, A.; Kojima, H.; Guo, C.; Oishi, A.; Yoshimura, N. Low-Dose-Rate, Low-Dose Irradiation Delays Neurodegeneration in a Model of Retinitis Pigmentosa. *Am. J. Pathol.* **2012**, *180*, 328–336, doi:10.1016/j.ajpath.2011.09.025.

11. Chen, M.; Huang, Q.; Xu, W.; She, C.; Xie, Z.G.; Mao, Y.T.; Dong, Q.R.; Ling, M. Low-dose X-ray irradiation promotes osteoblast proliferation, differentiation and fracture healing. *PLoS One* **2014**, *9*, doi:10.1371/journal.pone.0104016.

12. Velegzhaninov, I.O.; Ermakova, A. V.; Klokov, D.Y. Low dose ionizing irradiation suppresses cellular senescence in normal human fibroblasts. *Int. J. Radiat. Biol.* **2018**, *94*, 825–828, doi:10.1080/09553002.2018.1492167.

13. Cho, Y.H.; Kim, S.Y.; Woo, H.D.; Kim, Y.J.; Ha, S.W.; Chung, H.W. Delayed numerical chromosome aberrations in human fibroblasts by low dose of radiation. *Int. J. Environ. Res. Public Health* **2015**, *12*, 15162–15172, doi:10.3390/ijerph121214979.

14. Smith, R.W.; Mothersill, C.; Hinton, T.; Seymour, C.B. Exposure to low level chronic radiation leads to adaptation to a subsequent acute X-ray dose and communication of modified acute X-ray induced bystander signals in medaka (Japanese rice fish, Oryzias latipes). *Int. J. Radiat. Biol.* **2011**, *87*, 1011–1022, doi:10.3109/09553002.2011.587861.

15. Rozhko, T. V.; Badun, G.A.; Razzhivina, I.A.; Guseynov, O.A.; Guseynova, V.E.; Kudryasheva, N.S. On the mechanism of biological activation by tritium. *J. Environ. Radioact.* **2016**, *157*, 131–135, doi:10.1016/j.jenvrad.2016.03.017.

16. Planel, H.; Soleilhavoup, J.P.; Tixador, R.; Richoilley, G.; Conter, A.; Croute, F.; Caratero, C.; Gaubin, Y. Influence on cell proliferation of background radiation or exposure to very low, chronic γ radiation. *Health Phys.* **1987**, *52*, 571–578, doi:10.1097/00004032-198705000-00007.

17. Kovalchuk, O.; Arkhipov, A.; Barylyak, I.; Karachov, I.; Titov, V.; Hohn, B.; Kovalchuk, I. Plants experiencing chronic internal exposure to ionizing radiation exhibit higher frequency of homologous recombination than acutely irradiated plants. *Mutat. Res. - Fundam. Mol. Mech. Mutagen.* **2000**, *449*, 47–56, doi:10.1016/S0027-5107(00)00029-4.

18. Ha, H.J.; Subburaj, S.; Kim, Y.S.; Kim, J.B.; Kang, S.Y.; Lee, G.J. Molecular characterization and identification of calnexin 1 as a radiation biomarker from tradescantia BNL4430. *Plants* **2020**, *9*, doi:10.3390/plants9030387.

19. Zaka, R.; Vandecasteele, C.M.; Misset, M.T. Effects of low chronic doses of ionizing radiation on antioxidant enzymes and G 6 PDH activities in Stipa capillata (Poaceae)., doi:10.1093/jxb/erf041.

20. Luckey, T. Ionizing Radiation Promotes Protozoan Luckey, T. (1986). Ionizing Radiation Promotes Protozoan Reproduction - PubMed. Retrieved March 26, 2020, from Radition Research website: https://pubmed.ncbi.nlm.nih.gov/3097750/Reproduction - PubMed Available online: https://pubmed.ncbi.nlm.nih.gov/3097750/ (accessed on Mar 26, 2020).

21. Conter, A.; Dupouy, D.; Planel, H. Effects of Dose Rate on Response of Synechococcus lividus to Very Low Doses of Chronic γ Radiation: Influence of Enzymatic Equipment of Starting CellsEffects of Dose Rate on Response of Synechococcus lividus to Very Low Doses of Chronic g Radiation: Influence of Enzymatic Equipment of Starting Cells. *Radiat. Res.* **1986**, *105*, 379, doi:10.2307/3576693.

22. Robertson, K.L.; Mostaghim, A.; Cuomo, C.A.; Soto, C.M.; Lebedev, N.; Bailey, R.F.; Wang, Z. Adaptation of the Black Yeast Wangiella

dermatitidis to Ionizing Radiation: Molecular and Cellular Mechanisms. *PLoS One* **2012**, *7*, e48674, doi:10.1371/journal.pone.0048674.
